# Cell Polarization: A Comparative Cell Biology and Immunological View

**DOI:** 10.1155/2000/70801

**Published:** 2000

**Authors:** Miguel Vicente-Manzanares, Francisco Sánchez-Madrid

**Affiliations:** Servicio de Inmunología Hospital Universitario de la Princesa Universidad Autónoma de Madrid c/Diego de León 62 Madrid 28006 Spain

## Abstract

Cell polarization and the establishment of functionally specialized domains play a pivotal role in many cellular processes such as vectorial transport of molecules, cell division and differentiation, directional movement of the cells in a chemotactic gradient and activation of the immune response. Cell polarization is a complex phenomenon, in which the interplay among cell cytoskeletal components, extra- and intracellular signals and organelle and membrane reorganization is crucial to achieve a correct cell shape change. The intracellular machinery needed for cell polarization has been elucidated in several well-established models, including yeast, epithelial, neuronal and germ-line cells. Cells of the immune system also polarize in response to extracellular cues, but many of the intracellular signals that control cell polarization and the role of genes with a well-defined function in other polarization processes are still unknown. In this review, recent advances in the study of leukocyte polarization are examined highlighting the similarities and differences with other models of cell polarization. The extracellular signals which direct cell polarization, the signal transduction pathways involved as well as the role of cell polarization in the development of the immune response are discussed.


*Developmental Immunology*, 2000, Vol. 7(2-4), pp. 51-65
Reprints available directly from the publisher
Photocopying permitted by license only

© 2000 OPA (Overseas Publishers Association)
N.V. Published by license under the
Harwood Academic Publishers imprint,
part of the Gordon and Breach Publishing Group.
Printed in Malaysia

### Cell Polarization: A Comparative Cell Biology and Immunological View

MIGUEL VICENTE-MANZANARES and FRANCISCO SÁNCHEZ-MADRID*

*Servicio de Inmunología, Hospital Universitario de la Princesa, Universidad Autónoma de Madrid, c/ Diego de León 62, 28006-Madrid, Spain*

Cell polarization and the establishment of functionally specialized domains play a pivotal role in many cellular processes such as vectorial transport of molecules, cell division and differentiation, directional movement of the cells in a chemotactic gradient and activation of the immune response. Cell polarization is a complex phenomenon, in which the interplay among cell cytoskeletal components, extra- and intracellular signals and organelle and membrane reorganization is crucial to achieve a correct cell shape change. The intracellular machinery needed for cell polarization has been elucidated in several well-established models, including yeast, epithelial, neuronal and germ-line cells. Cells of the immune system also polarize in response to extracellular cues, but many of the intracellular signals that control cell polarization and the role of genes with a well-defined function in other polarization processes are still unknown. In this review, recent advances in the study of leukocyte polarization are examined highlighting the similarities and differences with other models of cell polarization. The extracellular signals which direct cell polarization, the signal transduction pathways involved as well as the role of cell polarization in the development of the immune response are discussed.

### 1. INTRODUCTION

Cell polarization is a fundamental feature of many cell types which must display and maintain specialized cytoplasmic and membrane-associated domains in order to perform specific tasks, including differentiation, cell growth and division, migration, transmission of stimuli, or the development of the immune response.

Over the past twenty years, much effort has been put into elucidating the extracellular cues as well as the signaling machinery that regulate the acquisition of a polarized phenotype. From yeast to highly differentiated and specialized cells such as neurons or lymphocytes, all of them share the intrinsic capability to become polarized (Fig. 1), and a common signaling machinery highly conserved through evolution (Drubin and Nelson, 1996). Cell polarization plays different functional roles depending upon the cell lineage and developmental stage. Hence, polarization is intrinsic to the reproductive cycle of yeast cells (Madden and Snyder, 1998), whereas a polarized phenotype is essential for functional processes in neurons such as neurite extension, growth and stimulus transmission (Higgins et al., 1997).

The role of polarization in cells from the immune system has not been so well studied. Besides its critical role in migration, cell polarization also plays a

* Please address all correspondence to Francisco Sánchez-Madrid, Servicio de Inmunología, Hospital Universitario de la Princesa, c/ Diego de León 62, 28006-Madrid, Spain. (Tel. +34 91 5202307; Fax. +34 91 3092496, e-mail: fsanchez@hlpr.insalud.es)

pivotal role in cognate immune interactions between effector lymphocytes and antigen-presenting cells (APC) (Kupfer and Singer, 1989; Sanchez-Madrid and del Pozo, 1999).

### 2. FROM YEAST TO LEUKOCYTE POLARITY: ESTABLISHMENT OF TWO MORPHOLOGICAL POLES

*Saccharomyces cerevisiae* cells usually display a round shape, but become polarized twice in their life cycle. During the vegetative cycle, yeast develop a budding protrusion from which the daughter cell will form. This bud will emerge in one site or another depending on the haploid or diploid nature of the cell. The formation of this budding cap is a tightly regulated phenomenon with a complex signalling machinery involved in its formation and development. The process of bud emergence consists of three steps: (1) Bud site selection: The small G protein Bud1, which encodes a Ras-related small GTP binding protein, has been demonstrated to play a key role in electing the place of bud emergence, as overexpression of either an activated mutant or a dominant negative form of the protein results in random budding. This protein is in turn regulated by two other genes, the GTPase activating protein (GAP) Bud2 and the guanosine exchange factor (GEF) Bud5 (Bender and Pringle, 1989; Park et al., 1993). (2) Assembly of the budding complex: Once the place of bud growth has been elected, an array of proteins is recruited to this site. These include the yeast homologue of Cdc42, which regulates actin assembly through the Wiskott-Aldrich syndrome protein yeast homologue Bee1/Las17 (Li, 1997) and the novel actin nucleation factor Pca-1 (Lechler and Li, 1997). Several gene products can be found at the budding tip of the cell, including Bem1 (Leeuw et al., 1995), an adapter protein which interacts with Ste20, the homologue of mammalian $p65^{PAK}$ (Manser et al., 1994) and Bni1, a homologue of mammalian formins (Evangelista et al., 1997), which is involved in morphogenesis and cytokinesis and could be the common mediator by which small GTP binding proteins, such as Cdc42, can stimulate actin polymerization. (3) Actin polymerization, polarized growth and *de novo* cell wall synthesis: The gene products described above control actin polymerization and the formation of a proper bud, which will eventually become a daughter cell, with a requirement for cell wall biosynthesis, a process which involves other related genes, such as the Rho1 protein, a close homologue of the mammalian RhoA gene, which regulates $\beta(1\rightarrow3)$-glucan synthase, an essential enzyme in cell wall biosynthesis (Drgonová et al., 1996; Qadota et al., 1996).

A similar process involving the same mediators occurs when a cell senses a mating pheromone gradient secreted by a mating phenotype-matched cell under adverse environmental conditions. In this case, the site selection machinery is replaced by a cluster of occupied pheromone-receptor pairs (Jackson et al., 1991), where they form a complex that enables actin polymerization and the extension of a mating projection, which will reach the mating partner and, after membrane fusion will give birth to a diploid zygote.

The polarized shape developed by *Saccharomyces* during bud or mating projection is strikingly similar to that of migrating leukocytes. When a leukocyte starts migration, extends a front protrusion (leading edge), where actin polymerization processes are very active (Mitchinson and Cramer, 1996), and retracts the microtubule-organizing center (MTOC) to the rear trailing edge, termed uropod (Ratner et al., 1997). Some cell adhesion receptors as well as cytoskeletal components are redistributed to the uropod, whereas chemosensory receptors, such as chemokine receptors, cluster at the cell leading edge (Sanchez-Madrid and del Pozo, 1999; Serrador et al., 1999). Nevertheless, bud extension and uropod formation similarities are not so obvious when examined at the molecular level. The main feature of the budding protrusion is a well-defined actin cap in the budding area. By contrast, uropod extension by migrating leukocytes is characterized by reduced polymerized actin, which concentrates at the cell leading edge, where the cell is continuously extending prospective lamellae and pseudopods enriched in chemosensory receptors (Sullivan et al., 1984; Mackay et al., 1991; Nieto et al., 1997; Serrador et al., 1999;). In this regard, it is worth

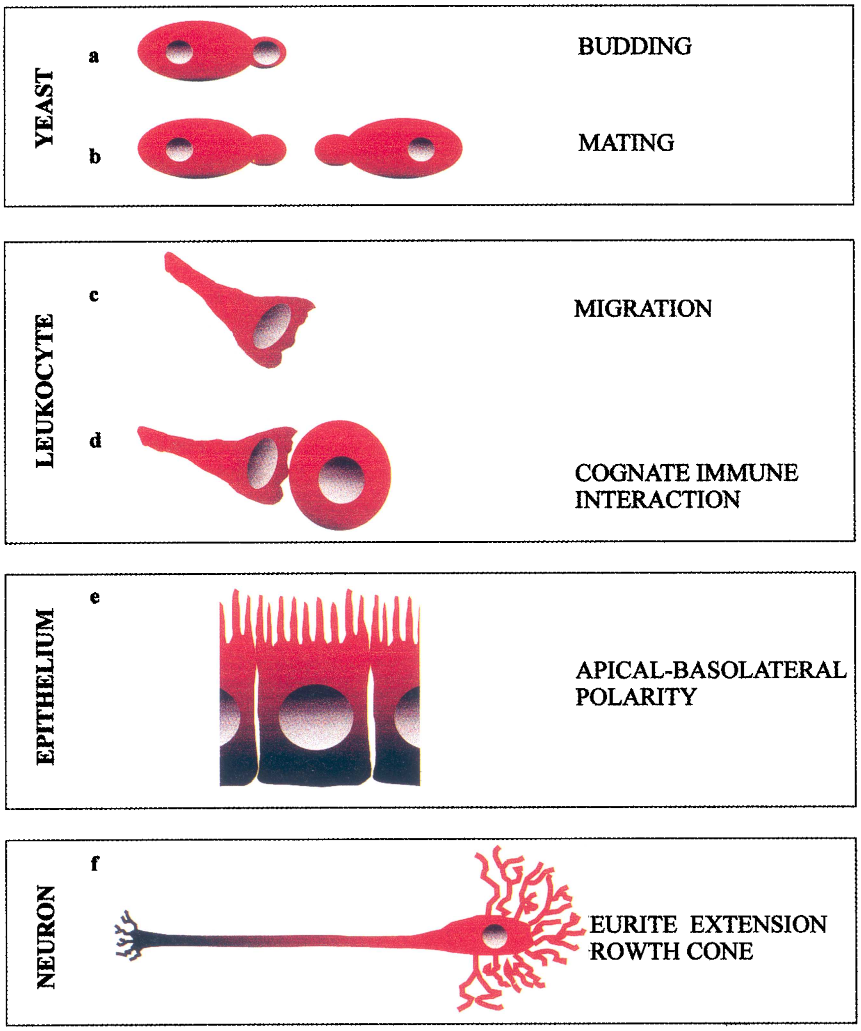

FIGURE 1 Polarization in different cell lineages. A schematic view of polarization in different cell types is shown. Yeast cell polarization during budding (a) and pheromone-induced mating (b). Chemoattractant-induced motile phenotype in leukocytes (c), and polarization during cognate immune interactions (d). Epithelial cell with apical and basolateral domains (e). Highly polarized neuron displaying neurite and axonal domains (f) (see Color Plate I at the back of this issue)

noting that there is a close resemblance between process extension during yeast mating and the generation of lamellae during chemotactic responses in both neutrophils and lymphoid cells (Nieto et al., 1997; Servant et al., 1999). Thus, the leading edge of migrating leukocytes and the directional pole through which Saccharomyces senses the chemotactic gradient would play a similar exploratory role, as it has been proposed (Sanchez-Madrid and del Pozo, 1999).

### 3. DYNAMICS OF THE CYTOSKELETON: ROLES OF ACTIN AND TUBULIN

Epithelial cells and neurons share a very stable polarization, which is retained as an intrinsic feature along their cell cycle whereas yeast or leukocyte polarization is a transient phenomenon, in which the cell becomes polarized from a normally round-shaped morphology. In the latter case, cell polarization is a

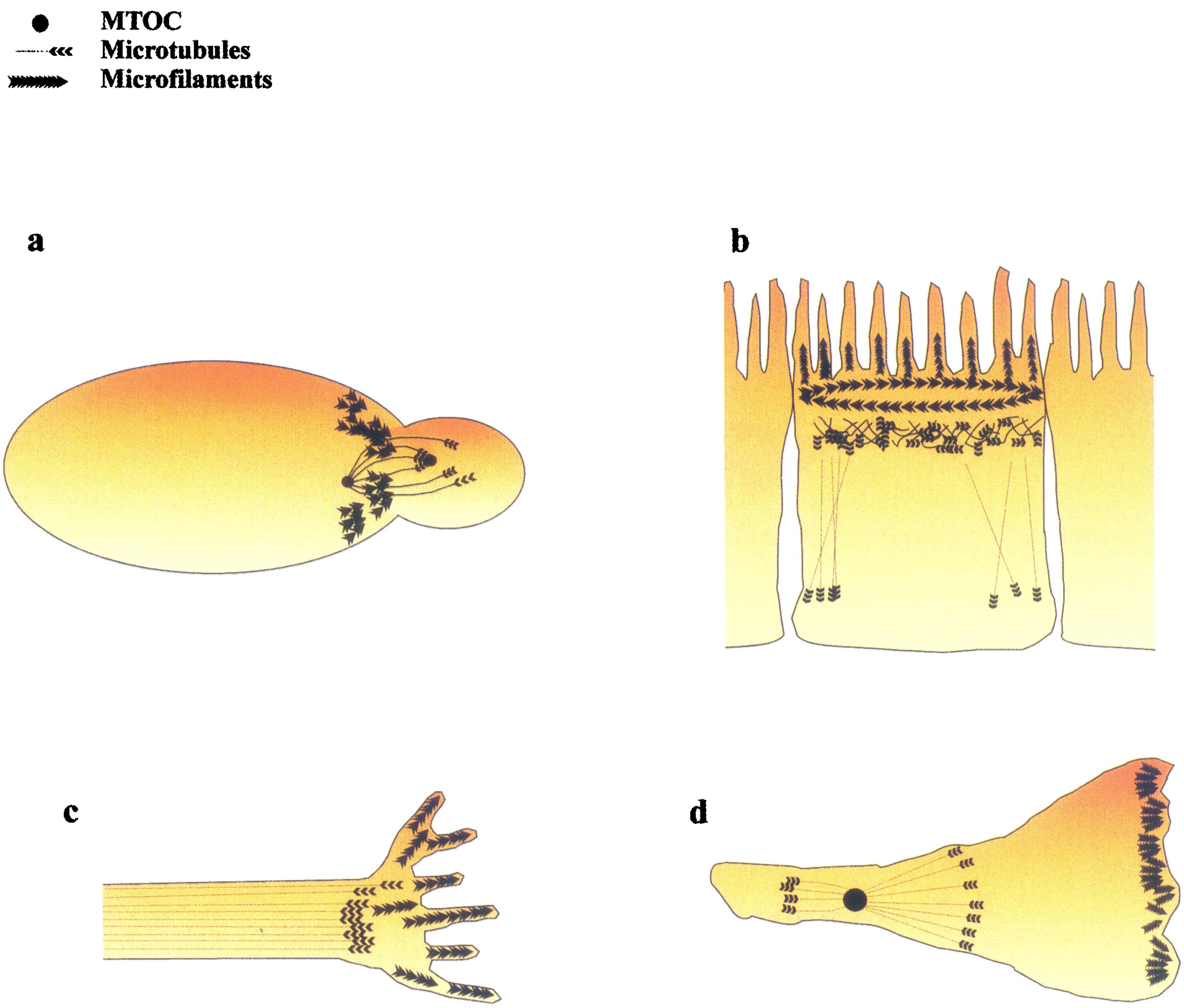

FIGURE 2 Cytoskeletal distribution in polarized cells. Microfilamentous and microtubular cytoskeletal reorientation in (a) Budding yeast, (b) Epithelial cells, (c) Neuron growth cone and (d) Migrating leukocyte (see Color Plate II at the back of this issue)

response that enables the cell to perform a specific and temporal task. The development of cell asymmetry relies greatly on the reorganization of the cytoskeleton. The role of the actin and tubulin cytoskeleton in the development of cell polarity has been investigated in different well-established models of cell polarity (Fig. 2).

ACTIN. Actin is a very active component of budding protrusions in yeast. It can be found forming patches in the cortex and very concentrated at the budding site. Actin cables starting in the bud run along the cell's length maintaining the morphology of the cell (Mulholland et al., 1994). Consistent with its important role in cell polarization, different mutations in actin genes result in defects in bud formation, growth arrest and the formation of huge cells unable to divide (Novick and Botstein, 1985). Actin dynamics is very active during yeast growth. Its localization is dependent on cell-cycle dependent kinases (CDKs), and during budding, the bud becomes a preferential place for actin polymerization and clustering (Kilmartin and Adams, 1984).

The establishment of leukocyte and fibroblast polarity involves the sequential adhesion of the cell to the substratum, formation of a front membrane protrusion supported by active actin polymerization, generation of contractile forces driven by myosin motors and rear detachment to allow cell migration (Lauffenburger and Horwitz, 1996; Serrador et al., 1999). Fibroblast adhesion to the substratum is very strong, with the formation of specialized structures at the cell-substratum contact area, which comprise clustered integrins and adapter proteins, which act as nucleation sites for actin bundles called stress fibers. The strong adhesion results in slow migration speed. On the other hand, the lack of well-defined focal adhesions and stress fibers at the cell-substratum contact area is a characteristic feature of migrating lymphocytes that may account for a decreased adhesion which in turn allows enhanced motility (Palecek et al., 1997).

Actin polymerization in neurons resembles closely what can be observed in leukocytes. The growth cone can be considered as the prospective leading edge of the neuron, and actin polymerization is most active there. Actin polymerization involves regulators like monomer-binding proteins (profilin, thymosin β4), and barbed-end capping proteins, such as Arp2/3, vasodilator-stimulated phosphoproteins (VASP) and Mena (Suter and Forscher, 1998). The balance between growth and the maintenance of the cell morphology is carried out by actin retrograde flow, which is regulated by myosin motors. Inhibition of myosin results in halted retrograde actin flow and the growth of filopodia at the leading edge (Lin et al., 1996).

In epithelial cells, actin dynamics are governed by the need of establishing two well defined and functionally distinct plasma membrane domains. Cell adhesion as well as cell-cell contacts guide actin polymerization at the sites of the contact, which may play a role in the formation of two poles by retaining or directing protein sorting specific of the cell adhesion sites (Clark and Brugge, 1995). The establishment of cell-substrate and cell-cell contacts also results in the formation of the apical face of the epithelial cell. There, actin polymerization is very active, though the adapter proteins that are located to the apical pole are different to those in the basolateral pole, resulting in a different composition of the actin cytoskeleton. The actin filaments assembled at the apical pole play a pivotal role in the formation of microvilli, specialized hair-like protrusions, which confer absorptive properties to polarized epithelia (Heinztzelman and Mooseker, 1992).

The regulation of actin dynamics by signaling proteins will be described in detail below.

TUBULIN. Analysis of the positioning of the microtubule network within budding yeast cells suggested that it could be involved in generation of buds or at least in bud site selection (Adams and Pringle, 1984). A better understanding of bud formation revealed that microtubule location in the budding tip is one of the last steps in bud formation, and it is not essential in the establishment of this structure (Snyder et al., 1991). Instead, it regulates subsequent phenomena such as nuclear segregation into the daughter cell (Page and Snyder, 1993).

The retraction of the microtubule-organizing center to the uropod of migrating lymphocytes has been described (Ratner et al., 1997; Serrador et al., 1999). The MTOC does not appear to support uropod formation, but it is likely involved in the maintenance of the cell deformability to allow extravasation (Ratner et al., 1997). However, the MTOC is translocated from the uropod to the intercellular contact area during antigen-specific immune recognition in both T helper-antigen presenting cells and killer cell-target cell interactions. This has been proposed as a mechanism of reorientation of the secretory apparatus in order to mediate the polarized secretion of either cytokines or lytic vesicles (Kupfer and Singer, 1989).

Epithelial cell polarity is also characterized by a dramatic reorganization of the microtubules. A dense patch of randomly oriented microtubules can be observed at the apical pole of the cell, whereas other bundles with the growing tips oriented towards the basolateral pole are located parallel to cell-cell lateral contacts. Microtubule localization in epithelial cells is critical as it regulates the sorting of apical and basolateral proteins by reorientation of the secretory apparatus (Drubin and Nelson, 1996).

Microtubules perform similar functions in neurons. A dense pack of microtubules can be observed in the center of the growth cone, supporting the structure and acting as carriers to provide the elements necessary for axonal growth (Baas and Brown, 1997; Hirokawa et al., 1997). Dynamic studies have revealed that microtubules are not steady structures, rather they are continuously growing and retracting, a phenomenon called dynamic instability. Microtubule motors, including members of the dynein families are likely regulatory candidates for such phenomena. There is a complex interplay between the microtubular and the microfilamentous system in growth cones during process extension. The most likely possibility is that tension generated by actomyosin motors in the periphery of the growth cone pulls forward the central microtubular region. In this regard, microtubule-associated protein 2c (MAP2c) has been shown to interact both with actin and tubulin, thus providing a link between the two cytoskeletal systems (Cunningham et al., 1997). However, microtubule advancement due to attenuation of retrograde flow cannot be ruled out.

### 4. ROLE OF PROTEIN SORTING IN THE ESTABLISHMENT AND MAINTENANCE OF CELL POLARITY

How is polarity maintained? As we have discussed before, the cytoskeleton plays a key role in the acquisition as well as in the maintenance of polarity. One key feature of a polarized cell is the different composition of the two poles of the cell, not only at the level of asymmetric redistribution of the cytoskeleton, but also in terms of the plasma membrane proteins and lipids (Mays et al., 1994). This distribution seems to be regulated by targeted vesicle transport from the Golgi apparatus, and by retention through association to the cytoskeleton. Motifs regulating protein targeting to one pole of the cell or the other is a very active field of study. Tyrosine- and dileucine- based domains are present in most basolateral-redistributed proteins, as demonstrated in MDCK (Madine-Darby canine kidney) cells (Matter and Mellman, 1994). This seems to be a very conserved mechanism in the development of different tissues. In this regard, overexpression of basolateral-targeted genes in neurons using virus-based vectors guides the gene product to the dendrites (Higgins et al., 1997), although the study of endogenous dendritic proteins such as the transferrin receptor rules out these signals being the only involved in dendritic sorting (West et al., 1997). Parallelism between apical and axonal sorting is not so obvious. In epithelium, GPI-anchored proteins appear to be targeted to the apical membrane, but in neurons they do not follow any distribution pattern based on this structural glycolipid link. In this regard, other non-GPI-anchored proteins, which are expressed on the apical face of MDCK cells, are not redistributed to the axon when expressed in neurons (Hoop et al., 1995).

Leukocyte polarity is a much more transient phenomenon which depends upon extracellular stimuli. Therefore, in resting leukocytes most plasma proteins are not stably concentrated at any particular site of the plasma membrane. However, upon stimulation, a rapid redistribution of surface molecules occurs. Some receptors become concentrated at the cell leading edge, such as the urokinase plasminogen activator receptor (uPAR) (Estreicher et al., 1990), the integrin $\alpha_v\beta_3$ (Lawson and Maxfield, 1996) as well as receptors for fMLP in neutrophils (Sullivan et al., 1984) and chemokine receptors in lymphocytes (Nieto et al., 1997). Maintenance of such receptors in the cell leading edge requires the existence of a polarized recycling mechanism that has been demonstrated for $\alpha_v\beta_3$ integrin in crawling neutrophils (Lawson and Maxfield, 1996). Other adhesion molecules such as ICAM-1, –2, –3, CD43, CD44 and PSGL-1 become clustered at the uropod (Sanchez-Madrid and del Pozo, 1999). The existence of motifs involved in differential redistribution to the front or rear pole of the cell is currently under investigation. A role can be inferred for cytoskeleton-linker proteins such as ERM components, which are redistributed with ICAMs and CD44 to the cellular uropod (Serrador et al., 1997;

Tsukita et al., 1997), and thus could direct ICAMs to the rear pole.

During antigenic presentation, redistribution of proteins become crucial to achieve a correct effector function of lymphocytes. T cells interact with their target cells through the leading edge (Negulescu et al., 1996). Initial contact is followed by a complex cascade of events including TCR interaction with the peptide-MHC complex, accumulation of LFA-1 integrin to stabilize the interaction, and redistribution of proteins involved in the specific recognition such as the TCR and other signaling proteins such as PKC-θ, and the Src-kinases Lck and Fyn (Monks et al., 1997, 1998). These molecules are clustered in supramolecular activation clusters (SMAC) which are included in glycosphingolipid-cholesterol-enriched membrane domains (rafts) (Dustin and Shaw, 1999; Viola et al., 1999; Wulfing and Davis, 1998). Stabilization of the effector-target conjugate results in reorientation of the MTOC and the Golgi complex, which are undoubtedly involved in polarized secretion of effector molecules, such as cytokines (Podack and Kupfer, 1991).

### 5. EXTRACELLULAR CUES THAT INDUCE POLARITY: SIGNAL TRANSDUCTION

Cells such as neurons or epithelial cells develop intrinsic polarity, whereas in yeasts or leukocytes, polarization is dependent upon an environmental signal to exert a specific response. A number of genes have been implicated in initiating as well as in transmitting signals whose outcome will be cell polarity. Despite their specific role in each cell lineage, general outlines of protein function can be deduced from genetic analysis.

### Extracellular cues

In yeast, two polarization modes have been so far described, budding and mating (Fig. 1). In budding, the extracellular cue consists of an environmental circumstance of high nutrient concentration. On the other hand, hormone-induced mating yeast polarization occurs when the cell is in the proximity to a mating-matched cell and senses a gradient of the mating pheromone. In this situation, pheromone receptors, which belong to the seven-spanned transmembrane domains, G-protein coupled receptor family, cluster in the tip of the mating projection to direct growth along the pheromone gradient. The mating signal can be mimicked in pheromone receptor-deficient cells by activation of downstream effectors of pheromone receptors, but in this case the cells are unable to discriminate between mating partners (Jackson et al., 1991).

Leukocyte polarization closely resembles yeast mating. A number of signals have been shown to induce the acquisition of a polarized phenotype. Most of these signals are chemoattractant molecules belonging to the interleukin, chemokine or complement family, and many of them signal through seven spanned-transmembrane domains, G-protein coupled receptors (Baggiolini, 1998; Baggiolini et al., 1997; Rollins, 1997). The similarity comes from the fact that chemokine receptors also become redistributed to the cell leading edge while leukocytes are sensing a chemokine gradient (Nieto et al., 1997). In this regard, chemokine receptor polarization would be guiding the cell navigation through the chemoattractant gradient (Fig. 3). It is not unlikely that clusters of pheromone or chemoattractant receptors are guiding the cytoskeletal growth or reorientation to keep the cell growing or moving in the right direction.

Chemoattractant receptor redistribution is a controversial field of study, and seems to be a cell-lineage dependent phenomenon. In this regard, it has been described that GFP-cAR1 (cAR-1 is the primary receptor of cAMP in *Dyctiostelium*) is not redistributed to the cell leading edge in *Dyctiostelium* cells for which cAMP is a potent chemoattractant (Xiao et al., 1997). Instead, differential localization and activation of G proteins associated to chemoattractant receptors has been observed on the cell side that senses the chemoattractant stimulus (Jin et al., 2000; Parent et al., 1998) as well as the appearance of binding sites for pleckstrin homology domain-containing proteins (Parent and Devreotes, 1999) and the translocation of PH-containing proteins to the cell leading edge (Servant et al., 2000). A recent study on the localization

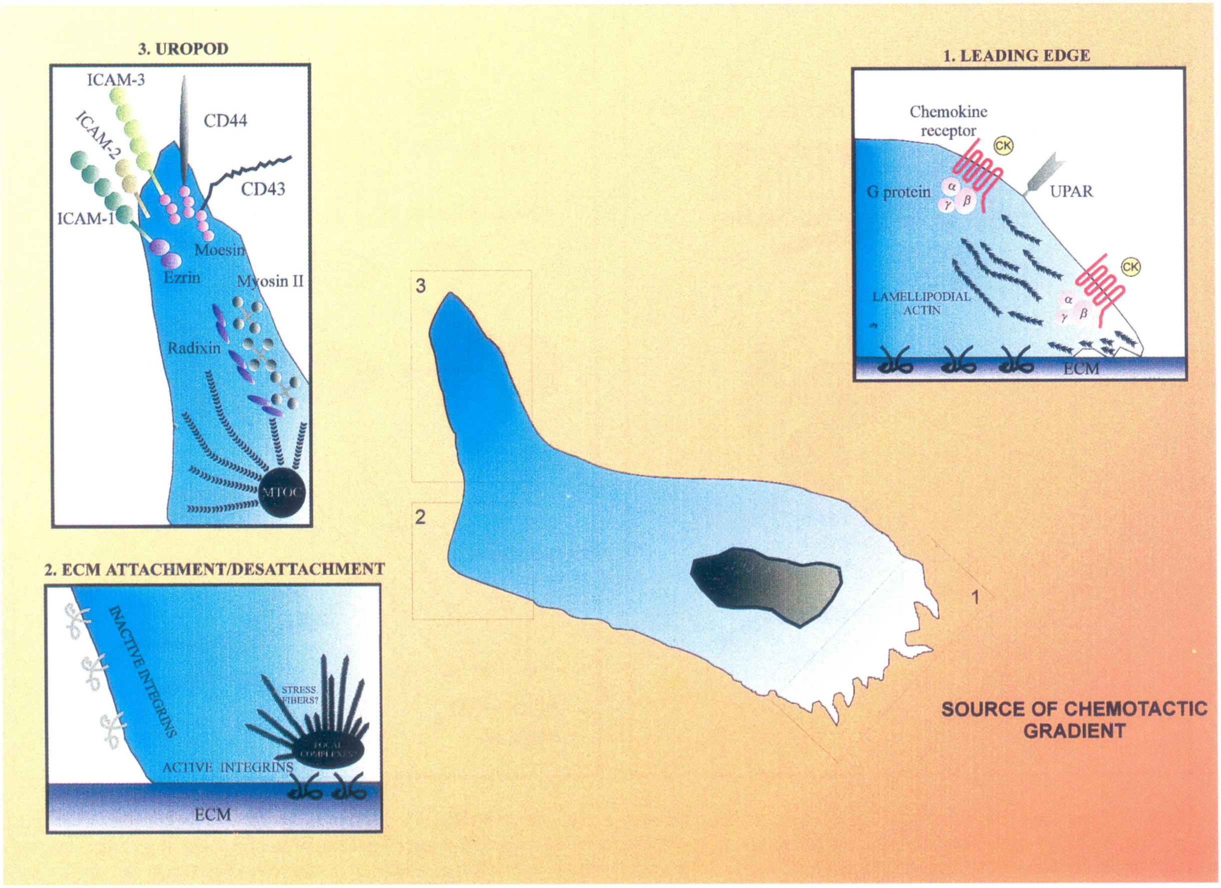

FIGURE 3 Leukocyte polarization, cytoskeletal and adhesion molecules redistribution and the establishment of leading edge and uropod poles. A schematic model of leukocyte polarization during navigation through a chemoattractant gradient is shown. High-detailed domains correspond to the leading edge (1), cell-extracellular matrix (ECM) rear contact area (2), and the rear pole (uropod) (3). Only cytoskeletal components and surface molecules with a role in cell-cell or cell-matrix interactions or in the perception of the chemotactic gradient are shown (see Color Plate III at the back of this issue)

of GFP-C5aR in a human neutrophilic leukemia cell line suggests that chemoattractant receptor redistribution could be explained in terms of membrane folding at the cell leading edge, which would imply a clustering of receptors without an increase of the number of receptors per membrane area (Servant et al., 1999).

### Signaling through chemoattractant and pheromone receptors

Signals induced by chemoattractant receptors comprise a large number of adaptor and signaling intermediates. We will review only those signals whose importance in establishing or maintaining cell polarity has been demonstrated.

Upon pheromone triggering, a signaling complex is formed downstream of the pheromone receptor. Receptor activation induces the dissociation of the $G_{\beta\gamma}$ subunits from $G_{\alpha i}$, and their association to Ste20, the yeast homologue of $p65^{PAK}$, thus linking pheromone signaling with the activation of mitogenic cascades. Upon activation, Cdc42 is also recruited to the mating projection, where it interacts with Ste20 (Peter et al., 1996), and it might regulate the association of

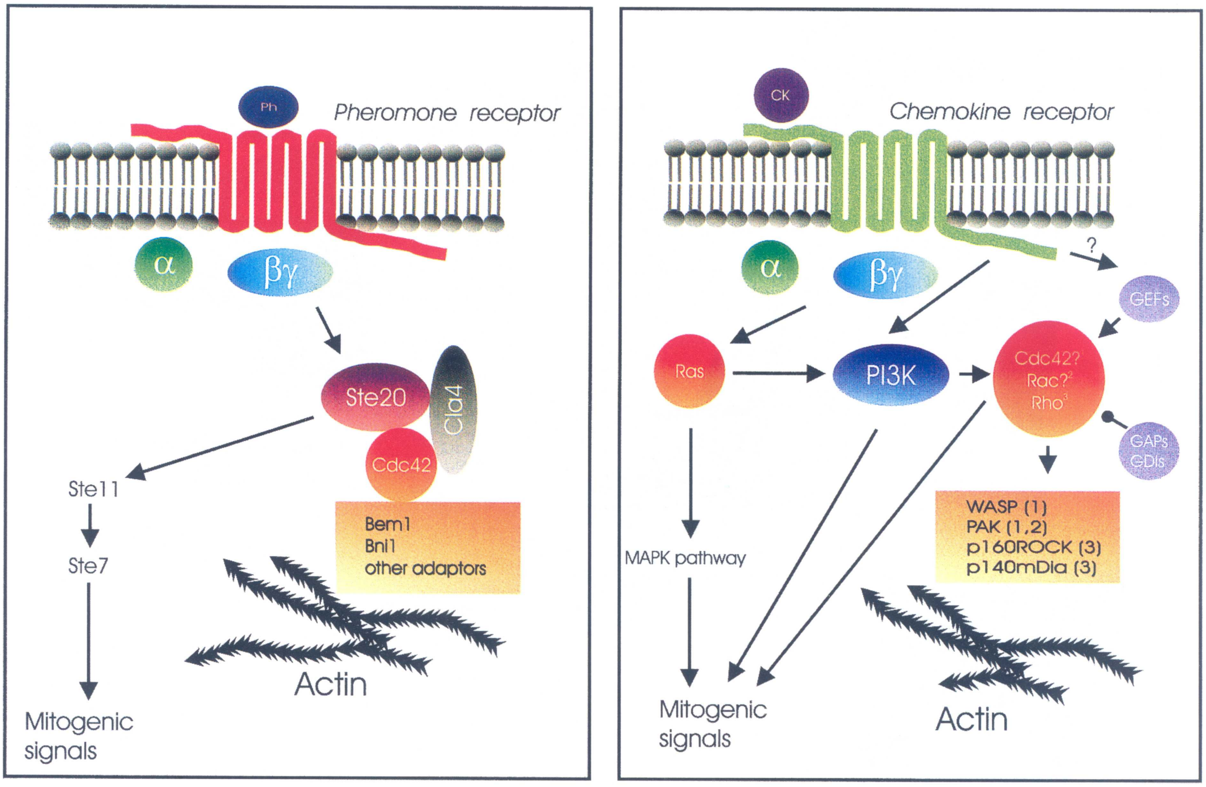

FIGURE 4 Signals induced by pheromone and chemoattractant receptors in yeast and leukocytes. Pheromone or chemokine binding to seven-spanned transmembrane domains, G-protein coupled receptors trigger the formation of a signaling complex associated to the cytoplasmic domain of the receptor. Signaling proteins with a prominent role in actin-redistribution and cell polarization are shown, whereas intermediates implicated in mitogenic pathways are only indicated (see Color Plate IV at the back of this issue)

adapter proteins such as Bni1 or Bem1 to the actin cytoskeleton, thus becoming a pivotal piece in the establishment of cell polarity. In agreement with this argumentation, all these molecules are found to interact with each other in budding or mating projections (Evangelista et al., 1997; Sheu et al., 1998).

Signals produced by chemokines binding to their receptors have been in the limelight for the last three years because of the capability of some chemokine receptors to act as correceptors for HIV (Littman, 1998). Chemokine triggering of its receptor is rapidly followed by $G_{\alpha i}$ dissociation from the βγ subunit, PLC activation and rises in intracellular calcium in a *pertussis* toxin-sensitive fashion. In addition, activation of other proteins, including RAFTK/ Pyk2 (Ganju et al., 1998), phosphatydil inositol 3-kinase (PI3K) (Turner et al., 1995; Turner et al., 1998), the JAK/STAT pathway (Mellado et al., 1998) and the small GTPase Rho (Bacon et al., 1998) has been reported.

The role of Rho family members in the reorganization of the actin cytoskeleton and in the morphology of the cell was first demonstrated in fibroblasts. Microinjection of activated mutants of Rho, Rac and Cdc42 caused profound changes in the actin distribution pattern, hence Cdc42 induced the formation of filopodia (Nobes and Hall, 1995), Rac induced membrane ruffling and the formation of protrusive lamellipodia (Ridley et al., 1992) and Rho generated stress fibers and promoted the formation of focal adhesions

(Ridley and Hall, 1992), where actin cables and integrin patches were found to interact through signaling/adaptor complexes (Burridge and Chrzanowska-Wodnicka, 1997; Clark and Brugge, 1995).

Although a hierarchy has been suggested and even demonstrated for fibroblasts, in which Cdc42 activated Rac and Rac activated Rho (Nobes and Hall, 1995), recent evidences point to antagonic roles of Rac and Rho GTPases in the control of the cell morphology and cell-substrate (Rottner et al., 1999; Sander et al., 1999). In this regard, it has been shown that Rac is involved in the formation of the cell leading edge through actin-based ruffling, initial sites of attachment to the substratum (focal complexes) and spreading (Rottner et al., 1999), whereas Rho plays a role in the consolidation of the attachment of the cell to the substratum by enlargement of the focal complexes, thereafter referred to as focal adhesions (Rottner et al., 1999) (Fig. 5). A direct role for Rho in the activation state of the integrins has been also postulated (Laudanna et al., 1996).

A direct role in polarization for members of the Rho subfamily has also been proposed. Fibroblasts migrating in a wound-healing assay acquire a polarized morphology. Cdc42 has been demonstrated to regulate the cell polarity through the control of correct reorientation of the cell leading edge, whereas Rac is essential to keep directional movement (Nobes and Hall, 1999).

In this regard, Arp2/3, a Cdc42 effector, has been recently shown to localize to the sites of actin nucleation at the cell's leading edge, thus suggesting a role of this signaling cascade in the establishment of two functional poles in migrating cells (Weiner et al., 1999).

In addition, a role has been ascribed to Cdc42 in T cell acquisition of polarity during antigen presentation (Stowers et al., 1995) and directional macrophage chemotaxis to CSF-1 (Allen et al., 1998), thus suggesting a key role for GTPases of the Rho subfamily not only in actin rearrangement but also in proper polarization and cell orientation.

The role of small GTP binding proteins of the Rho subfamily in stably polarized cells has also been addressed. In neurons, activation of Rho in growth cones causes collapse of the structure, which suggests a role for Rho in the regulation of actin polymerization and retrograde flow (Kozma et al., 1997). On the other hand, a regulatory role has been proposed for Rho GTPases in cadherin-dependent contacts in epithelial cell monolayers (Braga et al., 1997; Jou and Nelson, 1998).

Several molecules arose as putative downstream signaling intermediates linking Rho GTPases to the actin cytoskeleton. These included Wiskott-Aldrich syndrome protein (WASP) downstream of Cdc42 (Symons et al., 1996), $p65^{PAK}$ downstream of Rac (Sells et al., 1997) and Rho-associated kinase (Rho-kinase) downstream of Rho (Amano et al., 1997; Matsui et al., 1996). Taken together, the data reviewed suggest a common and highly conserved signaling mechanism that links extracellular cues with the cytoskeleton, regulating cell morphology.

### 6. POLARIZATION IN THE IMMUNE SYSTEM: HOMING, INFLAMMATION AND ANTIGENIC PRESENTATION

The main characteristic associated to lymphoid polarization is net cell movement. As described, chemotactic stimuli such as chemokines induce cell polarization, and the final outcome is cell chemotaxis following the chemotactic gradient. In this regard, chemokines and other chemotactic stimuli promote the acquisition of motor capability for which cell polarization seems to be an essential requirement. In addition, other accessory roles have been also proposed to lymphoid polarization and adhesion molecules redistribution, such as cooperative recruitment of lymphocytes (del Pozo et al., 1997).

Chemokines can be divided into two groups according with their role in haemostasis. Several chemokines, such as RANTES, MCP-1, MIP-1$\alpha$, MIP-1$\beta$ are produced mainly in inflammatory situations and recruit host defense cells to the site of inflammation. On the other hand, chemokines such as SDF-1$\alpha$, BCA-1/BLC or ELC are produced in lymphoid nodes and appear to play a prominent role in

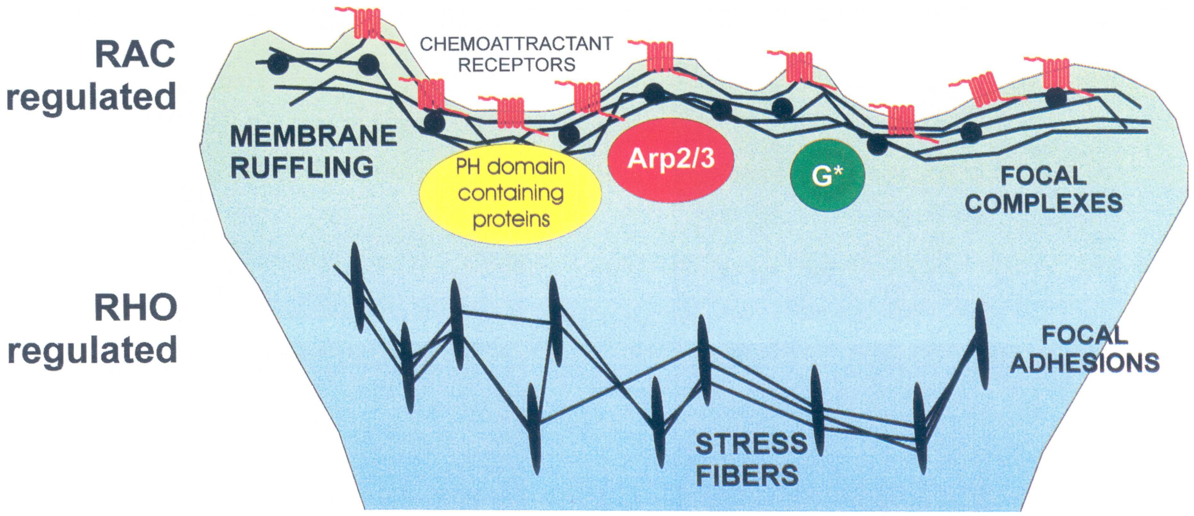

FIGURE 5 Localization of signaling intermediates and actin polymerization at the migrating cell's leading edge. Chemoattractant signaling induces the localization of signaling complexes, actin-polymerization machinery, and the coordinated activation of Rho and Rac GTPases to ensure the formation of actin structures needed for migration (see Color Plate V at the back of this issue)

lymphocyte homing and homeostasis (Baggiolini, 1998) (Mantovani, 1999).

Chemokines have been also involved in regulating the firm adhesion and arrest of rolling lymphocytes to blood vessel endothelium (Campbell et al., 1998). In this regard, the role of chemokines would be a dual one, in the first steps of recruitment to the inflammatory foci they would be promoting cell adhesion to the endothelial layer, and in later stages (after diapedesis), they would be directing the cell towards the inflammatory focus (Springer, 1994).

Cell positioning within the lymph node is a key feature in the development of lymphoid organs. The maintenance of well-defined areas is crucial for the establishment of cell-cell and cell-matrix interactions which ensure a proper development and maturation of the different lineages of cells involved in the genesis of the lymph node. In this regard, "systemic" chemokines such as SDF-1$\alpha$ and BCA-1/BLC seem to play a pivotal role in development and maturation of the immune system. Mice lacking the SDF-1 gene and those lacking its unique chemokine receptor CXCR4, share common abnormalities in the development of B lymphocytes along with other genetic defects, such as vascularization of the gastrointestinal tract and defects in the ventricular septum (Nagasawa et al., 1996; Tachibana et al., 1998; Zou et al., 1998). On the other hand, deletion of the BLR1/CXCR5 receptor (specific for BCA-1/BLC) results in aberrant architecture of lymph nodes and acute defects in homing capability and positioning of lymphocytes within the lymph node (Föster et al., 1996).

Very recently, a role has been demonstrated for the chemokine receptor CCR7 in the organization of the primary immune response (Forster et al., 1999). Taken together, these data strongly suggest a role for this kind of chemokines in motility within the lymph node leading to a proper architecture of the organ. Interested readers are pointed to the work of Melchers et al. (1999) for an excellent review on the role of chemokines in the organization of the humoral immune response.

Another situation where lymphoid polarization plays a key role is antigenic presentation. It has been shown that T cells develop enhanced sensitivity to antigen presentation on the cell advancing front, demonstrating that calcium-dependent T cell polarization and orientation of the leading edge to the APC are critical elements in successful antigen presentation (Negulescu et al., 1996). In NK-target cell-cell interactions, some molecules, which are also clustered in the leading edge of migrating cells, are found in the killer-target contact area (Nieto et al., 1998).

As for translocation of signaling machinery to the leading edge of migrating cells, molecules involved in signal transduction such as PKC-θ and RAFTK/Pyk2 have been found in the contact area of cytotoxic lymphocytes and NK cells with target (Monks et al., 1998 ; Sancho et al., 2000), further demonstrating the role of effector cell leading edge positioning towards the target cell to allow successful contact and proper effector function.

### 7. CONCLUDING REMARKS

The study of evolutionary divergent cells in the acquisition of a polarized morphology reveals striking similarities in terms of adapter molecules, cytoskeletal components and asymmetric membrane composition. What is most exciting is that common steps of cell polarization exist, and the signaling intermediates involved are highly conserved from low eukaryotic cells such as yeasts to more differentiated cells, such as lymphocytes or neurons.

Many functional roles of cell polarization have been already elucidated, but both the extracellular cues that induce and control polarity as well as the intracellular signal transduction machinery involved are extremely active fields of study. The discovery of new signals and links between molecules involved in cell polarity coming from different cellular models will allow us to get a clear-cut view of this exciting field.

### *Acknowledgements*

The critical reading of the manuscript is gratefully acknowledged to Dr. Carlos Cabañas, María Yáñez-Mó, Mercedes Rey and Juan M. Serrador. Grants SAF99/0034-CO1 and 2FD97-0680-CO2-O2 from the Spanish Ministerio de Educación y Ciencia have supported this work.

### *References*

Adams, A., and Pringle, J. (1984). Relationship of actin and tubulin distribution to bud growth in wild-type and morphogenetic mutant *Saccharomyces Cerevisiae*. J. Cell Biol. *98*, 934–945.

Allen, W. E., Zicha, D., Ridley, A. J., and Jones, G. E. (1998). A role for Cdc42 in macrophage chemotaxis. J. Cell Biol. *141*, 1147–1157.

Amano, M., Chihara, K., Kimura, K., Fukata, Y., Nakamura, N., Matsuura, Y., and Kaibuchi, K. (1997). Formation of actin stress fibers and focal adhesions enhanced by Rho-kinase. Science *275*, 1308–1311.

Baas, P. W., and Brown, A. (1997). Slow axonal transport- the polymer transport model. Trends Cell Biol. *7*, 380–384.

Bacon, K. B., Schall, T. J., and Dairaghi, D. J. (1998). RANTES activation of phospholipase D in Jurkat T cells: recruitment of GTP-binding proteins Arf and RhoA. J. Immunol. *160*, 1894–1900.

Baggiolini, M. (1998). Chemokines and leukocyte traffic. Nature *392*, 565–568.

Baggiolini, M., Dewald, B., and Moser, B. (1997). Human chemokines: an update. Annu. Rev. Immunol. *15*, 675–705.

Bender, A., and Pringle, J. R. (1989). Multicopy suppression of the cdc24 budding defect in yeast by CDC42 and three newly identified genes including the ras-related gene RSR1. Proc. Natl. Acad. Sci. USA *86*, 9976–9980.

Braga, V. M. M., Machesky, L. M., Hall, A., and Hotchin, N. A. (1997). The small GTPases Rho and Rac are required for the establishment of cadherin-dependent cell-cell contacts. J. Cell Biol. *137*, 1421–1431.

Burridge, K., and Chrzanowska-Wodnicka, M. (1997). Focal adhesion assembly. Trends Cell Biol. *7*, 342–347.

Campbell, J. J., Hedrick, J., Zlotnik, A., Siani, M. A., Thompson, D. A., and Butcher, E. C. (1998). Chemokines and the arrest of lymphocytes rolling under flow conditions. Science *279*, 381–384.

Clark, E. A., and Brugge, J. S. (1995). Integrins and signal transduction: the road taken. Science *268*, 233–239.

Cunningham, C. C., Leclerc, N., Flanagan, L. A., Lu, M., Janmey, P. A., and Kosik, K. S. (1997). Microtubule-associated protein 2c reorganizes both microtubules and microfilaments into distinct cytological structures in an actin-binding protein-280-deficient melanoma cell line. J. Cell Biol. *136*, 845–857.

del Pozo, M. A., Cabanas, C., Montoya, M. C., Ager, A., Sanchez-Mateos, P., and Sanchez-Madrid, F. (1997). ICAMs redistributed by chemokines to cellular uropods as a mechanism for recruitment of T lymphocytes. J. Cell Biol. *137*, 493–508.

Drgonová, J., Drgón, T., Tanaka, K., Kollár, R., Chen, G. C., Ford, R. A., Chan, C. S., Takai, Y., and Cabib, E. (1996). Rho1p, a yeast protein at the interface between cell polarization and morphogenesis. Science *272*, 277–279.

Drubin, D. G., and Nelson, J. W. (1996). Origins of cell polarity. Cell *84*, 335–344.

Dustin, M. L., and Shaw, A. S. (1999). Costimulation: building an immunological synapse. Science *283*, 649–650.

Estreicher, A., Muhlhauser, J., Carpentier, J. L., Orci, L., and Vasalli, J. D. (1990). The receptor for urokinase type plasminogen activator polarizes expression of the protease to the leading edge of migrating monocytes and promotes degradation of enzyme inhibitor complexes. J. Cell Biol. *111*, 783–792.

Evangelista, M., Blundell, K., Longtine, M. S., Chow, C. J., Adames, N., Pringle, J. R., Peter, M., and Boone, C. (1997).

Bni1p, a yeast formin linking cdc42p and the actin cytoskeleton during polarized morphogenesis. Science *276*, 118–122.

Föster, R., Mattis, A. E., Kremmer, E., Wolf, E., Brem, G., and Lipp, M. (1996). A putative chemokine receptor, BLR1, directs B cell migration to defined lymphoid organs and specific compartments of the spleen. Cell *87*, 1037–1047.

Forster, R., Schubel, A., Breitfeld, D., Kremmer, E., Renner-Muller, I., Wolf, E., and Lipp, M. (1999). CCR7 coordinates the primary immune response by establishing functional microenvironments in secondary lymphoid organs. *Cell.* 99:23-33.

Ganju, R. K., Brubaker, S. A., Meyer, J., Dutt, P., Yang, Y., Qin, S., Newman, W., and Groopman, J. E. (1998). The α-chemokine, stromal cell-derived factor-1α, binds to the transmembrane G-protein-coupled CXCR-4 receptor and activates multiple signal transduction pathways. J. Biol. Chem *273*, 23169–23175.

Heinztzelman, M., and Mooseker, M. (1992). Assembly of the intestinal brush border cytoskeleton. Annu. Rev. Cell Biol. *26*, 93–122.

Higgins, D., Burack, M., Lein, P., and Banker, G. (1997). Mechanisms of neuronal polarity. Curr. Opin. Neurobiol. *7*, 599–604.

Hirokawa, N., Terada, S., Funakoshi, T., and Takeda, S. (1997). Slow axonal growth-the subunit transport model. Trends Cell Biol. *7*, 384–388.

Hoop, M. J. d., Poser, C. v., Lange, C., Ikonen, E., Hunziker, W., and Dotti, C. G. (1995). Intracellular routing of wild type and mutated polymeric immunoglobulin receptor in hippocampal neurons in culture. J. Cell Biol. *130*, 1447–1459.

Jackson, C. L., Konopka, J. B., and Hartwell, L. H. (1991). S. cerevisiae alpha pheromone receptors activate a novel signal transduction pathway for mating partner discrimination. Cell *67*, 389–402.

Jackson, C. L., Konopka, J. B., and Hartwell, L. H. (1991). *S. Cerevisiae* α-pheromone receptor activate a novel signal transduction pathway for mating partner discrimination. Cell *67*, 389–402.

Jin, T., Zhang, N., Long, Y., Parent, C.A., and Devreotes, P.N. (2000). Localization of the G protein betagamma complex in living cells during chemotaxis. *Science.* 287:1034-1036.

Jou, T. S., and Nelson, W. J. (1998). Effects of regulated expression of mutant RhoA and Rac1 small GTPases on the development of epithelial (MDCK) cell polarity. J. Cell Biol. *142*, 85–100.

Kilmartin, J. V., and Adams, A. E. M. (1984). Structural rearrangements of tubulin and actin during the cell cycle of the yeast *Saccharomyces.* J. Cell Biol. *98*, 922–933.

Kozma, R., Sarner, S., Ahmed, S., and Lim, L. (1997). Rho family GTPases and neuronal growth cone remodelling: relationship between increased complexity induced by Cdc42Hs, Rac1, and acetylcholine and collapse induced by RhoA and lysophosphatidic acid. Mol. Cell. Biol. *17*, 1201–1211.

Kupfer, A., and Singer, S. J. (1989). Cell biology of cytotoxic and helper T cell functions: immunofluorescence microscopic studies of single cells and cell couples. Annu. Rev. Immunol. *7*, 309–337.

Laudanna, C., Campbell, J. J., and Butcher, E. C. (1996). Role of Rho in chemoattractant-activated leukocyte adhesion through integrins. Science *271*, 981–983.

Lauffenburger, D. A., and Horwitz, A. F. (1996). Cell migration: a physically integrated molecular process. Cell *84*, 359–369.

Lawson, M. A., and Maxfield, F. R. (1996). Ca2+ and calcineurin-dependent recycling of an integrin to the front of migrating lymphocytes. Nature *377*, 75–78.

Lechler, T., and Li, R. (1997). In vitro reconstitution of cortical actin assembly sites in budding yeast. J. Cell Biol. *138*, 95–103.

Leeuw, T., Fourest-Lieuvin, A., Wu, C. L., Chenevert, J., Clark, K., Whiteway, M., Thomas, D. Y., and Leberer, E. (1995). Pheromone response in yeast: association of Bem1p with proteins of the MAP kinase cascade and actin. Science *270*, 1210–1213.

Li, R. (1997). Beel, a yeast protein with homology to Wiscott-Aldrich syndrome protein, is critical for the assembly of cortical actin cytoskeleton. J. Cell Biol. *136*, 649–658.

Lin, C.-H., Espreafico, E. M., Mooseker, M. S., and Forscher, P. (1996). Myosin drives retrograde F-actin flow in neuronal growth cones. Neuron *16*, 769–782.

Littman, D. R. (1998). Chemokine receptors: keys to AIDS pathogenesis? Cell *93*, 677–679.

Mackay, D. A., Kuskel, J. R., and Wilkinson, P. C. (1991). Studies of chemotactic factor-induced polarity in human neutrophils. Lipid mobility, receptor distribution and time-sequence of polarization. J. Cell Sci. *100*, 473–479.

Madden, K., and Snyder, M. (1998). Cell polarity and morphogenesis in budding yeast. Annu. Rev. Microbiol. *52*, 687–744.

Manser, E., Leung, T., Salihuddin, H., Zhao, Z. S., and Lim, L. (1994). A brain serine/threonine protein kinase activated by Cdc42 and Rac1. Nature *367*, 40–46.

Mantovani, A. (1999). The chemokine system: redundancy for robust outputs. Immunol. Today *20*, 254–257.

Matsui, T., Amano, M., Yamamoto, T., Chihara, K., Nakafuku, M., Ito, M., Nakano, T., K Okaw, Iwamatsu, A., and Kaibuchi, K. (1996). Rho-associated kinase, a novel serine/threonine kinase, as a putative target for the small GTP-binding protein Rho. EMBO J. *15*, 2208–2216.

Matter, K., and Mellman, I. (1994). Mechanisms of cell polarity: sorting and transport in epithelial cells. Curr. Opin. Cell Biol. *6*, 545–554.

Mays, R. W., Beck, K. A., and Nelson, W. J. (1994). Organization and function of the cytoskeleton in polarized epithelial cells: a component of the protein sorting machinery. Curr. Opin. Cell Biol. *6*, 16–24.

Melchers, F., Rolink, A.G., and Schaniel, C. (1999). The role of chemokines in regulating cell migration during humoral immune responses. *Cell.* 99:351-354.

Mellado, M., Rodriguez-Frade, J. M., Aragay, A., Real, G. d., Martin, A. M., Vila-Coro, A. J., Serrano, A., Jr., F. M., and Martinez-A, C. (1998). The chemokine monocyte chemotactic protein-1 (MCP-1) triggers Janus kinase 2, activation and tyrosine phosphorylation of the CCR2B receptor. J. Immunol. *161*, 805–813.

Mitchinson, T. J., and Cramer, L. P. (1996). Actin-based cell motility and cell locomotion. Cell *84*, 371–379.

Monks, C. R., Freiberg, B. A., Kupfer, H., Sciaky, N., and Kupfer, A. (1998). Three-dimensional segregation of supramolecular activation clusters in T cells. Nature *395*, 82–86.

Monks, C. R. F., Kupfer, H., Sciaky, N., and Kupfer, A. (1997). Selective modulation of protein kinase-0 during T cell activation. Nature *385*, 83–86.

Mulholland, J., Preuss, D., Moon, A., Wong, A., Drubin, D., and Botstein, D. (1994). Ultrastructure of the yeast actin cytoskeleton and its association with the plasma membrane. J. Cell Biol. *125*, 381–391.

Nagasawa, T., Hirota, S., Tachibana, K., Takakura, N., Nishikawa, S., Kitamura, Y., Yoshida, N., Kikutani, H., and Kishimoto, T. (1996). Defects of B-cell lymphopoiesis and bone-marrow myelopoiesis in mice lacking the CXC chemokine PBSF/SDF-1. Nature *382*, 635–638.

Negulescu, P. A., Krasieva, T. B., Khan, A., Kerschbaum, H. H., and Calahan, M. D. (1996). Polarity of T cell shape, motility and sensitivity to antigen. Immunity *4*, 421–430.

Nieto, M., Frade, J. M., Sancho, D., Mellado, M., Martinez, A. C., and Sanchez-Madrid, F. (1997). Polarization of chemokine receptors to the leading edge during lymphocyte chemotaxis. J. Exp. Med. *186*, 153–158.

Nieto, M., Navarro, F., Perez-Villar, J. J., del Pozo, M. A., Gonzalez-Amaro, R., Mellado, M., Frade, J. M., Martinez, A. C., Lopez-Botet, M., and Sanchez-Madrid, F. (1998). Roles of chemokines and receptor polarization in NK-target cell interactions. J. Immunol. *161*, 3330–3339.

Nobes, C., and Hall, A. (1995). Rho, Rac and Cdc42 GTPases regulate the assembly of multimolecular focal complexes associated with actin stress fibers, lamellipodia and filopodia. Cell *81*, 52–62.

Nobes, C. D., and Hall, A. (1999). Rho GTPases control polarity, protrusion, and adhesion during cell movement. J. Cell Biol. *144*, 1235–1244.

Novick, P., and Botstein, D. (1985). Phenotypic analysis of temperature-sensitive yeast actin mutants. Cell *40*, 405–416.

Page, B., and Snyder, M. (1993). Chromosome segregation in yeast. Annu. Rev. Microbiol. *47*, 201–231.

Palecek, S. P., Loftus, J. C., Ginsberg, M. H., Lauffenburger, D. A., and Horwitz, A. F. (1997). Integrin-ligand binding properties govern cell migration through cell-substratum adhesiveness. Nature *385*, 537–540.

Parent, C. A., Blacklock, B. J., Froehlich, W. M., Murphy, D. B., and Devreotes, P. N. (1998). G protein signaling events are activated at the leading edge of chemotactic cells. Cell *95*, 81–91.

Parent, C. A., and Devreotes, P. N. (1999). A cell's sense of direction. Science *284*, 765–770.

Park, H.-O., Chant, J., and Herskowitz, I. (1993). BUD2 encodes a GTPase-activating protein for Bud1/Rsr1 necessary for proper bud-site selection in yeast. Nature *365*, 269–274.

Peter, M., Neiman, A. M., Park, H.-O., vanLohuizen, M., and Herskowitz, I. (1996). Functional analysis of the interaction between the small GTP binding protein Cdc42 and the Ste20 protein kinase in yeast. EMBO J. *15*, 7046–7059.

Podack, E. R., and Kupfer, A. (1991). T-cell effector functions: mechanisms for delivery of cytotoxicity and help. Annu. Rev. Cell Biol. *7*, 479–504.

Qadota, H., Python, C. P., Inoue, S. B., Arisawa, M., Anraku, Y., Zheng, Y., Watanabe, T., Levin, D. E., and Ohya, Y. (1996). Identification of yeast Rho1p GTPase as a regulatory subunit of (1–3)-beta glucan synthase. Science *272*, 279–281.

Ratner, S., Sherrod, W. S., and Lichlyter, D. (1997). Microtubule retraction into the uropod and its role in T cell polarization and motility. J. Immunol. *159*, 1063–1067.

Ridley, A. J., and Hall, A. (1992). The small GTP -binding protein rho regulates the assembly of focal adhesions and stress fibers in response to growth factors. Cell *70*, 389–399.

Ridley, A. J., Paterson, H. F., Johnston, D., Diekmann, D., and Hall, A. (1992). The small GTP-binding protein Rac regulates growth factor-induced membrane ruffling. Cell *70*, 401–410.

Rollins, B. (1997). Chemokines. Blood *90*, 909–928.

Rottner, K., Hall, A., and Small, J.V. (1999). Interplay between Rac and Rho in the control of substrate contact dynamics. *Curr. Biol.* 12:640-648.

Sanchez-Madrid, F., and del Pozo, M. A. (1999). Leukocyte polarization in cell migration and immune interactions. EMBO J *18*, 501–511.

Sancho, D., Nieto, M., Llano, M., Rodríguez-Fernández, J.L., Tedejor, R., Avraham, S., Cabañas, C., López-Botet, M., and Sánchez-Madrid, F. (2000). The tyrosine kinase PYK-2/RAFTK regulates Natural killer (NK) cell cytotoxic response, and is translocated and activated upon specific target recognition and killing. *J. Cell Biol.* 149:1249-1262.

Sander, E.E., Klooster, J.P.t., Delft, S.v., Kammer, R.A.v.d., and Collard, J.G. (1999). Rac downregulates Rho activity: reciprocal balance between both GTPases determines cellular morphology and migratory behavior. *J. Cell Biol.* 147:1009-1022.

Sells, M. A., Knaus, U. G., Bagrodia, S., Ambrose, D. M., Bokoch, G. M., and Chernoff, J. (1997). Human p21-activated kinase (Pak1) regulates actin organization in mammalian cells. Curr. Biol. *7*, 202–210.

Serrador, J. M., Alonso-Lebrero, J. L., del Pozo, M. A., Furthmayr, H., Schwartz-Albiez, R., Calvo, J., Lozano, F., and Sanchez-Madrid, F. (1997). Moesin interacts with the cytoplasmic region of intercellular adhesion molecule-3 and is redistributed to the uropod of T lymphocytes during cell polarization. J Cell Biol *138*, 1409–23.

Serrador, J. M., Nieto, M., and Sanchez-Madrid, F. (1999). Cytoskeletal rearrangement during migration and activation of T lymphocytes. Trends Cell Biol. *9*, 228–232.

Servant, G., Weiner, O. D., Neptune, E. R., Sedat, J. W., and Bourne, H. R. (1999). Dynamics of a chemoattractant receptor in living neutrophils during chemotaxis. Mol. Biol. Cell *10*, 1163–1178.

Servant, G., Weiner, O.D., Herzmark, P., Balla, T., Sedat, J.W., and Bourne, H.R. (2000). Polarization of chemoattractant receptor signaling during neutrophil chemotaxis. *Science.* 287:1037-1040.

Sheu, Y. J., Santos, B., Fortin, N., Costigan, C., and Snyder, M. (1998). SpaZp interacts with cell polarity proteins and signaling components involved in yeast cell morphogenesis. Mol. Cell. Biol. *18*, 4053–4069.

Snyder, M., Gehrung, S., and Page, B. D. (1991). Studies concerning the temporal and genetic control of cell polarity in *Saccharomyces Cerevisiae*. J. Cell Biol. *114*, 515–532.

Springer, T. A. (1994). Traffic signals for lymphocyte recirculation and leukocyte emigration: the multistep paradigm. Cell *76*, 301–314.

Stowers, L., Yelon, D., Berg, L. J., and Chant, J. (1995). Regulation of the polarization of T cells toward antigen-presenting cells by Ras-related GTPase CDC42. Proc. Natl. Acad. Sci. U.S.A. *92*, 5027–5031.

Sullivan, S. J., Daukas, G., and Zigmond, S. H. (1984). Asymmetric distribution of the chemotactic peptide receptor on polymorphonuclear leukocytes. J. Cell Biol. *99*, 1461–1467.

Suter, D. M., and Forscher, P. (1998). An emerging link between cytoskeletal dynamics and cell adhesion molecules in growth cone guidance. Curr. Opin. Neurobiol. *8*, 106–116.

Symons, M., Derry, J. M. J., Karlak, B., Jiang, S., Lemahicu, V., McCormick, F., Francke, U., and Abo, A. (1996). Wiskott-Aldrich syndrome protein, a novel effector for the GTPase Cdc42Hs, is implicated in actin polymerization. Cell *84*.

Tachibana, K., Hirota, S., Iizasa, H., Yoshida, H., Kawabata, K., Kataoka, Y., Kitamura, Y., Matsushima, K., Yoshida, N., Nishikawa, S., Kishimoto, T., and Nagasawa, T. (1998). The chemokine receptor CXCR4 is essential for the vascularization of the gastrointestinal tract. Nature *393*, 591–595.

Tsukita, S., Yonemura, S., and Tsukita, S. (1997). E/R/M (Ezrin/Radixin/Moesin) family: from cytoskeleton to signal transduction. Curr. Op. Cell Biol. *9*, 70–75.

Turner, L., Ward, S. G., and Westwick, J. (1995). RANTES-Activated human T lymphocytes. A role for phosphoinositide 3-kinase. J. Immunol. *155*, 2437–2444.

Turner, S. J., Domin, J., Waterfield, M. D., Ward, S. G., and Westwick, J. (1998). The CC chemokine monocyte chemotactic peptide-1 activates both the class I p85/p110 phosphatidylinositol 3-kinase and the class II PI3K-C2a. J. Biol. Chem. *273*, 25987–25995.

Viola, A., Schroeder, S., Sakakibara, Y., and Lanzavecchia, A. (1999). T lymphocyte costimulation mediated by reorganization of membrane microdomains. Science *283*, 680–682.

Weiner, O.D., Servant, G., Welch, M.D., Mitchinson, T.J., Sedat, J.W., and Bourne, H.R. (1999). Spatial control of actin polymerization during neutrophil chemotaxis. *Nat. Cell Biol.* 1:75-81.

West, A. E., Neve, R. L., and Buckley, K. M. (1997). Identification of a somatodendritic targeting signal in the cytoplasmic domain of the transferrin receptor. J. Neurosci. *17*, 6038–6047.

Wulfing, C., and Davis, M. M. (1998). A receptor/cytoskeletal movement triggered by costimulation during T cell activation. Science *282*, 2266–2269.

Xiao, Z., Zhang, N., Murphy, D. B., and Devreotes, P. N. (1997). Dynamic distribution of chemoattractant receptors in living cells during chemotaxis and persistent stimulation. J. Cell Biol. *139*, 365–374.

Zou, Y. R., Kottmann, A. H., Kuroda, M., Taniuchi, I., and Littman, D. R. (1998). Function of the chemokine receptor CXCR4 in haematopoiesis and in cerebellar development. Nature *393*, 595–599.

